# ﻿Taxonomic status and nomenclature of *Tanacetumclusii* (Asteraceae, Asteroideae, Anthemideae), with comments on its distribution

**DOI:** 10.3897/phytokeys.251.141311

**Published:** 2025-01-29

**Authors:** Viktor O. Nachychko, Clemens Pachschwöll, Mihai Puşcaş, Ghizela Vonica, Gergely Király

**Affiliations:** 1 Department of Botany, Faculty of Biology, Ivan Franko National University of Lviv, 4, Hrushevskoho Str., Lviv 79005, Ukraine; 2 Department of Botany and Biodiversity Research, Faculty of Life Sciences, University of Vienna, 14, Rennweg, Vienna 1030, Austria; 3 Department of Taxonomy and Ecology, Faculty of Biology and Geology, Babeș-Bolyai University, 44, Republicii Str., Cluj-Napoca 400015, Romania; 4 A. Borza Botanic Garden, Babeş-Bolyai University, 42, Republicii Str., Cluj-Napoca 400015, Romania; 5 Emil G. Racoviță Institute, Babeș-Bolyai University, 5-7, Clinicilor Str., Cluj-Napoca 400006, Romania; 6 Museum of Natural History, Brukenthal National Museum, 1, Cetății Str., Sibiu 550160, Romania; 7 Institute of Natural Resources and Forest Management, University of Sopron, 4, Bajcsy-Zsilinszky Str., Sopron 9400, Hungary

**Keywords:** *Chrysanthemum*, distribution, nomenclature, *Pyrethrum*, taxonomy, typification

## Abstract

The paper provides nomenclatural and taxonomic accounts on *Tanacetumclusii*, a diploid species found in the Eastern Alps, the Carpathians, and the Dinarides, as well as comments on its current distribution. A careful examination of historic taxonomic literature showed that the combination *T.clusii* was first proposed by Kerner and predates a currently used much younger isonym by Soják (1871 vs. 1971). One specimen, a karyovoucher from WU, is designated here as an epitype for the illegitimate name *Pyrethrumclusii*, upon which *Chrysanthemumclusii*, the basionym of *T.clusii*, is based. This designation aims to avoid ambiguity in the taxonomic interpretation of its previously selected lectotype. Based on examining the original material, a voucher from SIB is designated as a lectotype of the synonymic name *Chrysanthemumsubcorymbosum*, a basionym of Tanacetumcorymbosumsubsp.subcorymbosum. The last name is the only correct one in the rank of subspecies when *T.clusii* is alternatively treated as a separate subspecies within *T.corymbosum* s.l. In addition, one specimen from SAMU is designated here as a neotype of Pyrethrumcorymbosumf.macrocephalum, a newly discovered heterotypic synonym of *T.clusii*. The analysis of the current species distribution showed that *T.clusii* is native to Switzerland (confirmed!), Italy, Austria, Slovenia, Croatia, Bosnia and Herzegovina, Hungary (confirmed!), Slovakia, Poland, Ukraine, and Romania. Although currently not accepted for Switzerland in literature, *T.clusii* could be confirmed for the Swiss canton of the Grisons. The same applies for Hungary where, apart from the Bükk Mts in literature, new localities from the Kőszeg and Mátra Mts are presented here. Additionally, the presence of *T.clusii* in Bulgaria, the Czech Republic, France, Greece, Montenegro and Turkey has not been confirmed and recorded mistakenly in different sources.

## ﻿Introduction

The genus *Tanacetum* L. is one of the most taxonomically complex and species-rich groups in the family Asteraceae Bercht. & J. Presl. It comprises around 160 species of mostly perennial herbs and subshrubs found in the circum-Mediterranean region, central and eastern Asia, and parts of northern America (Sonboli et al. 2012). According to the newest phylogenetic classification (Oberprieler et al. 2022) this genus belongs to the subtribe Anthemidinae Dumort. of the tribe Anthemideae Cass. belonging to the subfamily Asteroideae Lindl. Especially crucial within the genus *Tanacetum* is the *Tanacetumcorymbosum* group (Iamonico 2018; Pulišová et al. 2021). It comprises 4–5 microspecies or subspecies whose taxonomic classification is still inconsistent (Heywood 1976a, 1976b; Pulišová et al. 2021; POWO 2024). A member of this group is *T.clusii* (Kreutzer) A. Kern. Its taxonomic status and nomenclature still need to be clarified, mainly due to the absence of a detailed nomenclatural revision accompanied by a critical examination of the original material and the typification of the synonymic name *Chrysanthemumsubcorymbosum* Schur (Iamonico 2018).

Current taxonomic sources have considered this taxon as a subspecies, namely Tanacetumcorymbosumsubsp.subcorymbosum (Schur) Pawł. (Euro+Med 2024; POWO 2024). However, the recent study by Pulišová et al. (2021), based on a comprehensive overview of the karyological and morphological variability of the *T.corymbosum* group in Slovakia, supports elevating this taxon to the species rank. In the paper (Pulišová et al. 2021), the name *T.clusii* proposed by Soják (Chrtková-Žertová et al. 1971), is used for this species. We share the opinion of Pulišová et al. (2021) that this taxon, being diploid (2*n* = 2*x* = 18), deserves a species rank and must be separated from the closely related tetraploid (2*n* = 4*x* = 36) *T.corymbosum* (L.) Sch.Bip. Furthermore, a careful examination of historic taxonomic literature showed that there is an older isonym of *T.clusii* proposed by Kerner in 1871, which must be applied for this species. Additionally, considerable controversy regarding the species distribution has been identified in various contemporary sources. This inconsistency is evident in authoritative global (e.g., Hassler 2024; POWO 2024) and regional (e.g., Argenti et al. 2019; Euro+Med 2024) databases, floras and checklists, leading to potential future errors. Hence, a critical review and synthesis of the available information on *T.clusii*’s distribution are needed.

This paper aims to realize a detailed nomenclatural and taxonomic survey of *Tanacetumclusii*, focusing on the ascertainment of its current synonymy and the typification of the synonymic name *Chrysanthemumsubcorymbosum*, the basionym of the alternatively accepted subspecies name Tanacetumcorymbosumsubsp.subcorymbosum. It also seeks to clarify some aspects of the current species’ distribution.

## ﻿Materials and methods

This study is based on an examination of relevant floristic and taxonomic literature. Herbarium specimens relevant for this study (including original and type material) were also studied in the herbaria BP, BRNU, CHUR, CL, DE, LI, LW, MSNM, SAMU, SIB, W, and WU (herbarium acronyms are given according to Thiers 2024). In addition, images from several herbaria available online were also consulted (B, BR, E, H, L, NEU, O, P, PI, S, Z, ZT). A list of selected herbarium specimens of *Tanacetumclusii* examined is given in Appendix 1. The articles cited in the text and nomenclatural inquiries discussed here follow the "International Code of Nomenclature for algae, fungi, and plants" (Turland et al. 2018, herein ICN). In the nomenclatural and taxonomic section of this paper, an accepted name is presented in bold font, while synonyms are in regular font.

## ﻿Nomenclatural and taxonomic history of *Tanacetumclusii* and related taxa

Over the course of history, plants of *Tanacetumclusii* have been classified within different genera of the aster family, namely *Chrysanthemum* L., *Pyrethrum* Zinn, and *Tanacetum*. For the first time, they were described from the lower mountains and valleys of Austria and Pannonia (i.e., Lower Austria, Burgenland and adjacent Western Hungary; Stafleu 1967) by Clusius (1601, pg. 337), who proposed for them a polynomial “Tanacetum inodorum I”. Later on, Reichenbach (1831–1832, pg. 231) described this species as *Pyrethrumclusii* Fisch. ex Rchb., honoring Clusius and citing his “Tanacetum inodorum I” in its synonymy (Iamonico 2018). However, the name *P.clusii* has been published by Reichenbach ten years after Tausch proposed the same name (Tausch 1821, pg. 8, 12). In the synonymy of his *P.clusii*, Tausch (1821, pg. 12) cited “Tanacetum inodorum II”, a different taxon from the work of Clusius (1601), which should be associated with *Tanacetumcorymbosum* s.str. (Iamonico 2018). Consequently, *P.clusii* by Reichenbach (1831–1832) is a later homonym and therefore, illegitimate according to Art. 53.1 of the ICN. Nevertheless, this name has been used in many contemporary European floras (e.g., Dostál 1989; Tzvelev 1994), identification keys (e.g., Dostál and Červenka 1992; Chopyk and Fedoronchuk 2015), and checklists (e.g., Mosyakin and Fedoronchuk 1999). The final epithet of this illegitimate name can be re-used in different replacement names with the same type (ICN, Art. 58.1). For the first time, this re-use was effected by Kreutzer (1840, pg. 60, 219), who transferred *P.clusii* Fisch. ex Rchb. to the genus *Chrysanthemum* and named it *C.clusii* Kreutzer. Thereby, Kreutzer (1840) formally published a legitimate name at the species rank with the epithet “clusii” and priority dated 1840, which should be treated as a basionym for subsequent nomenclatural combinations based on illegitimate *P.clusii* Fisch. ex Rchb. (ICN, Art. 41.4 – for combinations published before 1953, and Art. 41.8(c) – for combinations published on or after 1953). The first such combination was published by Kerner (1871, pg. 159), who included Reichenbach’s species within the genus *Tanacetum* as *T.clusii*. Afterwards, Halácsy (1896, pg. 273) and then Dostál (1950, pg. 1603) considered that this taxon deserves only the infraspecific rank within the related species *Chrysanthemumcorymbosum* L. (=*T.corymbosum*). They proposed the following new combinations C.corymbosumvar.clusii (Kreutzer) Halácsy and C.corymbosumsubsp.clusii (Kreutzer) Dostál, respectively.

Alternatively, Schur (1859, pg. 146) described *Chrysanthemumsubcorymbosum* Schur from the Eastern Carpathians, which, as can be concluded from Schur’s original description and the lectotype designated in this study, referred to *Pyrethrumclusii* sensu Reichenbach (1831–1832). Consequently, *C.subcorymbosum* Schur is a later taxonomic synonym of *C.clusii* Kreutzer within the genus *Chrysanthemum*. Nevertheless, *C.subcorymbosum* has become a basionym for many combinations that were alternatively used later for this taxon.

In 1865, Kanitz was the first to propose the combination *Tanacetumsubcorymbosum* (Schur) Kanitz, transferring Schur’s species into the genus *Tanacetum* (Kanitz 1865, pg. 652). One year later, Schur (1866) published the combination *Pyrethrumsubcorymbosum* (Schur) Schur, classifying this species within the genus *Pyrethrum*. The last combination, in fact, is a correct name for the species *P.clusii* sensu Reichenbach (1831–1832) within the *Pyrethrum* genus (ICN, Art. 11.4). Subsequently, in the taxonomic literature of the 19^th^ and 20^th^ centuries, *Chrysanthemumsubcorymbosum* Schur, similarly to *P.clusii* Fisch. ex Rchb., was often given infraspecific status within what is now known as *Tanacetumcorymbosum* s.l., but under different generic names. For instance, Simonkai (1887, pg. 312) classified Schur’s taxon as a variety, Tanacetumcorymbosumvar.subcorymbosum (Schur) Simonk. Later on, Beck von Mannagetta (1893, pg. 1204) and Degen (1938, pg. 151) each published similar combinations at the variety rank within the genera *Chrysanthemum* and *Pyrethrum*, respectively, as Chrysanthemumcorymbosumvar.subcorymbosum (Schur) Beck and Pyrethrumcorymbosumvar.subcorymbosum (Schur) Degen. Hayek (1928–1931, pg. 652), generally following the above classification of Simonkai (1887), treated this taxon as a subvariety T.corymbosumsubvar.subcorymbosum (Schur) Hayek. In contrast, Pawłowski (Szafer and Pawłowski 1936, pg. 21) and Ujhelyi (1941, pg. 109) elevated its infraspecific rank to a subspecies, proposing the nomenclatural combinations Tanacetumcorymbosumsubsp.subcorymbosum (Schur) Pawł. and Chrysanthemumcorymbosumsubsp.subcorymbosum (Schur) Ujhelyi, respectively.

In 1891, Waisbecker (1891, pg. 28), apparently unaware of Reichenbach’s (1831–1832) and Schur’s (1859) taxa, described a new form, Pyrethrumcorymbosumf.macrocephalum Waisb. The last name became a basionym for P.corymbosumvar.macrocephalum (Waisb.) Borbás, published by Borbás (1898, pg. 503) seven years later, and after that was practically neglected in the taxonomic literature. According to the original diagnosis and the neotype designated in this study, P.corymbosumf.macrocephalum is fully comparable with *P.clusii* sensu Reichenbach (1831–1832) and with *Chrysanthemumsubcorymbosum* Schur, thus being their newly discovered heterotypic synonym.

Heywood (1976a, 1976b) clarified the taxonomic conception of *Tanacetumcorymbosum*, recognizing both *Chrysanthemumcorymbosum* and *Pyrethrumcorymbosum* under this species. He proposed treating the representatives of *Pyrethrumclusii* sensu Reichenbach (1831–1832) and *Chrysanthemumsubcorymbosum* Schur as a distinct subspecies within *T.corymbosum*. Unaware of the illegitimate status of Reichenbach’s *P.clusii*, Heywood (1976a, pg. 272) based a nomenclatural combination for the proposed subspecies on the latter. That should indeed be treated as the valid publication of a new combination based on *Chrysanthemumclusii*Kreutzer (1840), the earliest replacement name for *P.clusii* sensu Reichenbach (1831–1832). Thus, Heywood’s reference to Reichenbach (1831–1832) as the basionym author is a correctable error, and the name is to be cited as T.corymbosumsubsp.clusii (Kreutzer) Heywood (ICN, Art. 41.8(c)). “T.corymbosumsubsp.clusii (Fisch. ex Rchb.) Soó 1971” is an analogous name which appeared in Soó (1972, 1973, 1980), and seems to be an earlier one that could have priority. Although likely intended to be published in Soó (1971) where he dealt, among many others, with *Chrysanthemum* names, it is lacking there, as well as in other known papers of R. Soó (Anonymous 1973) published in 1971. Meanwhile, the citations of this name by Soó (1972, 1973) cannot be regarded as valid publications that could support its priority status as compared with T.corymbosumsubsp.clusii by Heywood (1976a). Therefore, the latter is the only validly published name, and its final epithet, “clusii”, has priority at the rank of subspecies from 1950 when it was first used in the combination Chrysanthemumcorymbosumsubsp.clusii (Kreutzer) Dostál (ICN, Arts 11.2, 11.4). In this context, the combination Tanacetumcorymbosumsubsp.subcorymbosum, proposed by Pawłowski (Szafer and Pawłowski 1936) for the same subspecies, has a priority over Heywood’s (1976a) proposal (1936 vs. 1950) and must be accepted as the only correct name (ICN, Art. 11.4). Therefore, the subspecies rank and the name T.corymbosumsubsp.subcorymbosum, are commonly used for this taxon in recent literature (e.g., Bartolucci et al. 2018, 2024; Iamonico 2018; Gilli et al. 2020; Euro+Med 2024; POWO 2024).

Recently, Pulišová et al. (2021), based on a comprehensive overview of the karyological and morphological variability of the *Tanacetumcorymbosum* group in Slovakia, have advocated for recognizing *T.corymbosum* s.str. (=T.corymbosumsubsp.corymbosum) and T.corymbosumsubsp.subcorymbosum (=*T.clusii*) as two separate species, rather than subspecies. For the latter taxon, Pulišová et al. (2021) used the name *T.clusii*, as proposed by Soják in Chrtková-Žertová et al. (1971). However, this name is a younger isonym of *T.clusii* by Kerner (1871), as previously mentioned. Thus, the correct name for the species is *T.clusii* (Kreutzer) A. Kern.

## ﻿Taxonomic treatment

### ﻿Typifications

#### ﻿ Tanacetum clusii

Taxon classificationPlantaeAsteralesAsteraceae

(Kreutzer) A. Kern., Oesterr. Bot. Z. 21: 159. 1871.

D026D0C3-D72F-5ACA-ABBD-8A6D71551327

**Figure 1.**
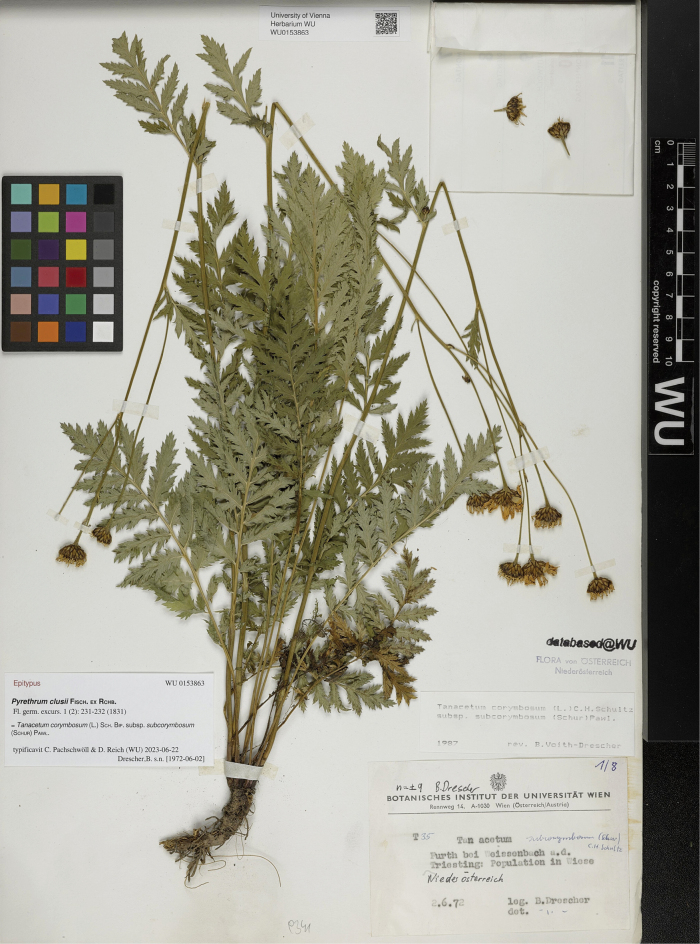
Epitype of *Pyrethrumclusii* Fisch. ex Rchb., nom. illeg. (WU [WU0153863]).

**Figure 2.**
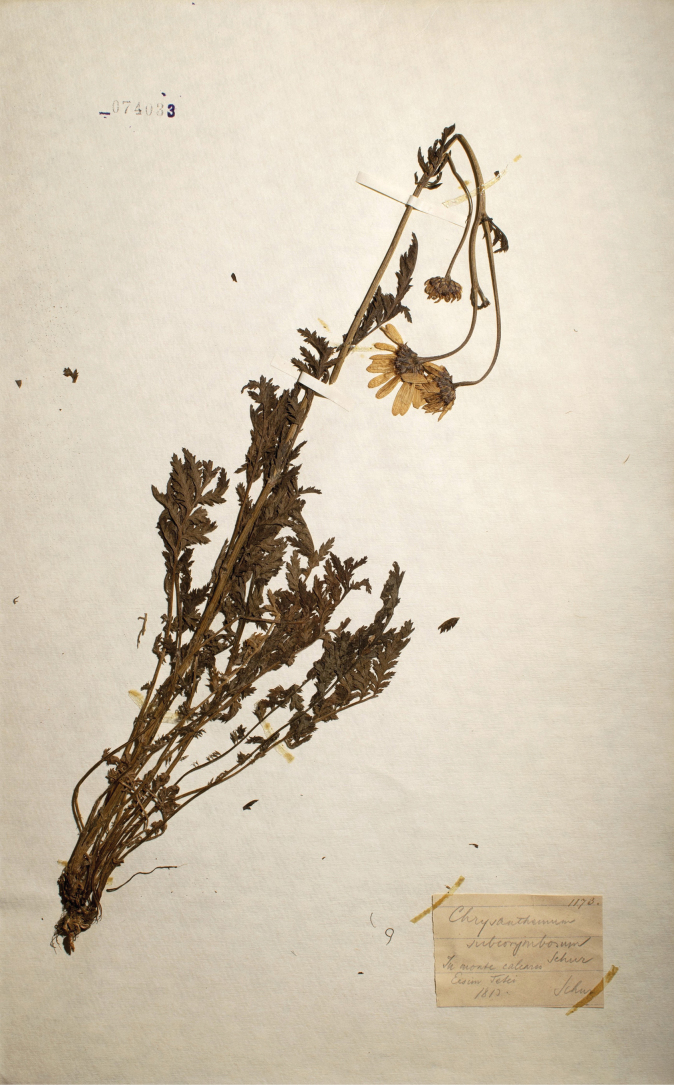
Lectotype of *Chrysanthemumsubcorymbosum* Schur (SIB [074033]).

**Figure 3.**
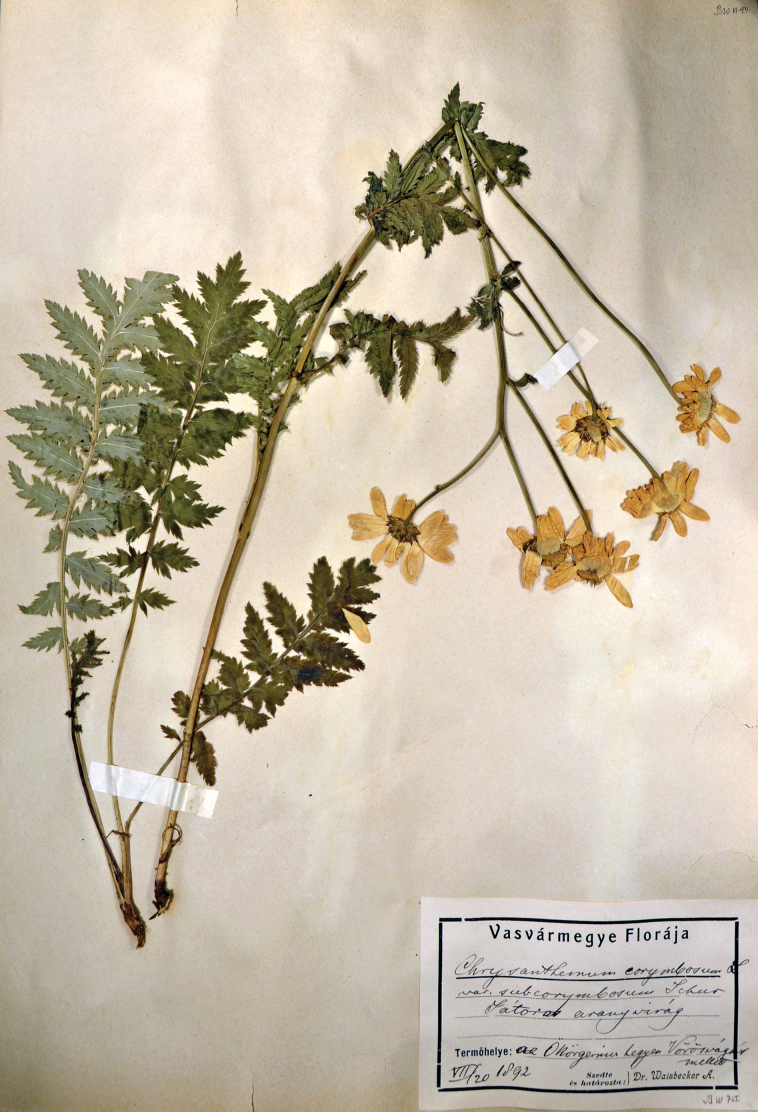
Neotype of Pyrethrumcorymbosumf.macrocephalum Waisb. (SAMU [Herb. Waisbecker]).

≡ Chrysanthemumclusii Kreutzer, Anthochronologion: 60, 219. 1840 [basionym].
≡ Pyrethrumclusii Fisch. ex Rchb., Fl. Germ. Excurs. 1(3): 231. 1831–1832 [replaced synonym; ICN, Art. 58.1], non Tausch, Index Seminum [Prague]: 8, 12 (no. 7). 1821, nom. illeg. Type: [Icon] “Tanacetum inodorum I” in Clusius, Rar. Pl. Hist.: 338. 1601 (lectotype, designated by Iamonico 2018, pg. 8 of 9 [image of lectotype available at http://biodiversitylibrary.org/item/14549#page/350/mode/1up]). AUSTRIA. Lower Austria: Furth bei Weissenbach a. d. Triesting, Population in Wiese, 2 Jun 1972, *B. Drescher s.n.* (epitype, designated here: WU [WU0153863!], Fig. 1 [image of epitype available at https://wu.jacq.org/WU0153863]; isoepitypes: WU [WU0153864!, WU0153865!, WU0153866!, WU0153867!, WU0153868!, WU0153869!, WU0153870!]).
≡ Chrysanthemumcorymbosumvar.clusii (Kreutzer) Halácsy, Fl. Niederösterr.: 273. 1896.
≡ Chrysanthemumcorymbosumsubsp.clusii (Kreutzer) Dostál, Květena ČSR: 1603. 1950.
≡ Tanacetumcorymbosumsubsp.clusii (Kreutzer) Heywood, Bot. J. Linn. Soc. 71: 272. 1976.
= Chrysanthemumsubcorymbosum Schur, Verh. Siebenb. Ver. Naturw. 10: 146. 1859. Type: ROMANIA. Harghita: In monte calcareo Ecsém Tetei [Piatra Ascuțită/Ecem], 1853, *F. Schur s.n.* (lectotype, designated here: SIB [074033!], Fig. 2; isolectotypes: CL [91643!], SIB [Herb. Fuss 25.312!; Herb. Ungar 42.521!]).
≡ Tanacetumsubcorymbosum (Schur) Kanitz, Magy. Birod. Termész. Visz. 3: 652. 1865.
≡ Pyrethrumsubcorymbosum (Schur) Schur, Enum. Pl. Transsilv.: 337. 1866.
≡ Tanacetumcorymbosumvar.subcorymbosum (Schur) Simonk., Enum. Fl. Tra­nssilv.: 312. 1887.
≡ Chrysanthemumcorymbosumvar.subcorymbosum (Schur) Beck, Fl. Nieder-Österreich 2(2): 1204. 1893.
≡ Tanacetumcorymbosumsubvar.subcorymbosum (Schur) Hayek, Repert. Spec. Nov. Regni Veg. Beih. 30(2): 652. 1931.
≡ Tanacetumcorymbosumsubsp.subcorymbosum (Schur) Pawł., Exsicc. (Fl. Polon.), ser. 2, 3: 21. 1936.
≡ Pyrethrumcorymbosumvar.subcorymbosum (Schur) Degen, Fl. Veleb. 3: 151. 1938.
≡ Chrysanthemumcorymbosumsubsp.subcorymbosum (Schur) Ujhelyi, Bor­básia 3: 109. 1941.
= Pyrethrumcorymbosumf.macrocephalum Waisb., Kőszeg Vid. Ed. Növ., ed. 2: 28. 1891. Type: AUSTRIA. Burgenland: Az Ökörgerincz hegyen Vörösvágás mellett [on the Ochsenriegel Mt close to Redlschlag], 20 Jul 1892, *A. Waisbecker AW 755* (neotype, designated here: SAMU [Herb. Waisbecker!], Fig. 3).
≡ Pyrethrumcorymbosumvar.macrocephalum (Waisb.) Borbás, Magy. Várm., Vas Várm.: 503. 1898.

### ﻿Nomenclatural notes

*Chrysanthemumclusii*, the basionym of *Tanacetumclusii*, is typified by the nomenclatural type of the illegitimate name *Pyrethrumclusii* Fisch. ex Rchb. (ICN, Art. 58.1). In 2018, Iamonico (2018) designated this nomenclatural type as a lectotype, selecting the illustration “Tanacetum inodorum I” from the work of Clusius (1601, pg. 338). However, the cited image, while providing a general view of the plant, lacks essential diagnostic features, such as the indumentum on the underside of leaves, the color of the involucral bract margins, as well as the ploidy level. For this reason, in some cases, this illustration can be identifiable to both *T.clusii* and *T.corymbosum* s.str., as well as their possible hybrids. The latter most likely occur in natural habitats common for both parental species (Pulišová et al. 2021, 2024: Supporting information). Thus, to avoid ambiguity in the taxonomic interpretation of the lectotype, an epitype of *P.clusii*, nom. illeg. is designated here (ICN, Art. 9.9). As the epitype, we select the specimen at WU [WU0153863], collected by B. Drescher in Lower Austria on 2 June 1972 (Appendix 1). The epitype was collected in the Northern Limestone Alps WSW of Baden, a region also visited by Clusius (Reichhardt 1866). This specimen, which morphologically represents *T.clusii*, additionally contains the label information about its ploidy (*n* = ± 9), being, in fact, the voucher specimen of unpublished karyological data by B. Drescher, a former PhD student of F. Ehrendorfer (1927–2023). The latter was interested in the karyology and evolution of *T.corymbosum* s.l. and had unpublished data on it in 1968 (Ehrendorfer 1970). Therefore, the epitype is undoubtedly *T.clusii*, considered to be diploid (2*n* = 2*x* = 18; Pulišová et al. 2021, 2024), and perfectly supports the lectotype designated by Iamonico (2018). It also corresponds with the lectotype of *Chrysanthemumsubcorymbosum*, designated here, confirming that *C.subcorymbosum* is an obvious taxonomic synonym of *T.clusii*. The last statement was a source of doubt till now because no original material of *C.subcorymbosum* has ever been examined (Iamonico 2018).

The epitype designated here is apparently part of a population sampling by B. Drescher for an unpublished karyological study. There are seven more specimens at the herbarium WU with identical labels, which were collected by B. Drescher at the same locality on 2 June 1972 (Appendix 1). The only substantial difference in label information between these eight specimens is their different numbers beginning with the letter “T” (means “Tanacetum”), i.e., T29 ([WU0153866]), T30 ([WU0153865]), T32 ([WU0153870]), T33 ([WU0153864]), T35 ([WU0153863]), T36 ([WU0153867]), T37 ([WU0153869]), and T38 ([WU0153868]). We consider these numbers to be the references to individuals of a single gathering (ICN, Art. 8.2 footnote, Art. 8 Note 1), which comprise the above specimens that, in turn, are duplicates (ICN, Art. 8.3, footnote). Therefore, the above duplicates of the selected epitype are considered here as isoepitypes (ICN, Art. 9.4, footnote).

The synonymic name *Chrysanthemumsubcorymbosum*, the basionym of the alternatively accepted subspecies name Tanacetumcorymbosumsubsp.subcorymbosum, was published in 1859 by Ferdinand Schur, a famous German-Austrian botanist and pioneer in the study of the flora of Transylvania (a historical region in present-day Romania) (Speta 1994). The respective work of Schur (1859) contains the result of his expedition throughout Transylvania, made from July 5^th^ to August 15^th^, 1853. This work is structured according to concrete excursions to different localities, for which the list of observed and collected plants is given, with descriptions of new taxa. Therefore, all the protologue data of new names are often located in different parts of Schur’s paper. In the case of *C.subcorymbosum*, besides its description given on page 146 under plant no. 358, there are provenance data on pages 119 and 142, i.e., “VII. Rodna, seine Gebirge und Umgegend, den 17. bis 23 Juli [VII. Rodna, its mountains and surroundings, 17 to 23 July]” (Schur 1859, pg. 119), “c) Der Koron oder Koronyis [c) Koron or Koronyis]” (Schur 1859, pg. 142). Moreover, on page 142, at the beginning of the respective species list for Koron [Mt Corongiş in the Rodna Mts], Schur wrote “Die hier verzeichneten Pflanzen verdanke ich der freundschaftlichen Mittheilung des Herrn Anton Czetz in Déés und Reckert in Nassod, in deren Sammlungen ich sie beobachtet habe [For the plants enumerated here, I owe to the friendly communication of Mr. Anton Czetz in Déés and Reckert in Nassod, in whose collections I observed them]”. It means the description of *C.subcorymbosum* mentioned in that list could be based only on Czetz and/or Reckert collections. In addition, on page 171, *C.subcorymbosum* is listed among plants collected and observed at Ecsem Teteje [Mt Piatra Ascuțită/Ecem] on 29 July 1853 (Schur 1859, pg. 160). This mention, which contains a reference to the species description published in this paper (“conf. VII, nro 358”) and followed by the text “Auf Kalksubstrat an sonnigen, grasigen Abhängen in einer Höhe von 4000’; blühend [On calcareous substrate on sunny, grassy slopes at an elevation of 4000’; blooming]”, can be treated as a second locality cited in the protologue. The results of this excursion to Mt Piatra Ascuțită/Ecem were also presented in a separate paper of Schur (1858), which was published one year before the description of *C.subcorymbosum* (Schur 1859; Speta 1994), and where the latter name nevertheless appeared as a nomen nudum.

Thus, assuming the above protologue data, the original material (ICN, Art. 9.4) of *Chrysanthemumsubcorymbosum* includes (1) any possible specimens associated with this taxon and collected by Czetz and/or Reckert at Mt Corongiş before 23 July 1853, (2) any respective specimens collected by Schur himself at Mt Corongiş (25 July 1853; Speta 1994, pg. 325) and Mt Piatra Ascuțită/Ecem (29 July 1853; Schur 1859, pg. 160) in the framework of his expedition made in 1853, (3) any other specimens associated with *C.subcorymbosum*, which were available to Schur prior to, or at the time of, preparation of his description, and can be found among his collections (uncited original material; ICN, Art. 9.4(a)). In this context, the mentioned specimens of Czetz and Reckert, as well as those collected by Schur at Mt Piatra Ascuțită/Ecem on 29 July 1853, which may be potentially revealed, are the syntypes (ICN, Art. 9.6). In case of specimens of Czetz and Reckert, it is because by linking collectors’ names with all the taxa in the list for Koron, and stating that these taxa were observed in private collections (see above), Schur (1859), in fact, cited concrete gatherings (ICN, Art. 40 Note 2). The citation of the concrete gathering was effected by Schur also by linking the date (“29 July 1853”; Schur 1859, pg. 160) with the locality (“Ecsem Teteje”; Schur 1859, pg. 160) and its details (Schur 1859, pg. 171), what has a place in the second case (ICN, Art. 40 Note 2). Such syntypes and their duplicates (isosyntypes) have a precedence for lectotype designation over the rest of the original material (ICN, Art. 9.12).

Looking for the original material of *Chrysanthemumsubcorymbosum*, we did not find any specimens of Czetz and Reckert suitable for lectotype selection. Meanwhile, by consulting herbaria where the collections of Schur are stored (Speta 1994; Nachychko 2014; Nachychko et al. 2024; Bionomia 2024), several specimens collected by Schur himself were detected at LW, CL, and SIB (Appendix 1). Among these specimens, those collected in July and August 1854 (LW [LW00208447 and LW00208452]), could scarcely be used for preparing the description of *C.subcorymbosum* since Schur had finished and submitted the manuscript of his paper (Schur 1859) apparently in the first half of 1854 (Speta 1994). Hence, we doubt that these two specimens comprise the original material of *C.subcorymbosum*, in contrast to the other five specimens collected during Schur’s expedition in 1853 and available for preparing his description (CL [91643], LW [LW00208453], SIB [074033; Herb. Ungar 42.521; Herb. Fuss 25.312]). The four specimens collected at Mt Piatra Ascuțită/Ecem in 1853 perfectly correspond to one of the syntypes cited in the protologue and are the best candidates for lectotype designation. These specimens stored at SIB and CL are obviously the parts of one gathering made on 29 July 1853, even though the collecting date and/or year are missing on the specimens’ labels. It should be noted here that three specimens at SIB, in contrast to one at CL, do not contain the original labels and were subscribed by different persons. At the same time, these specimens were evidently collected by Schur (Doltu and Schneider-Binder 1970). Thus, considering the above, the specimens at SIB together with the specimen at CL can be regarded as isosyntypes (ICN, Art. 9.6, Art. 9.4 footnote) being an apparent original material of *C.subcorymbosum*, irrespective of whether these specimens were seen by Schur preparing the description of this species (ICN, Art. 9.4(d)). Among these isosyntypes, the most representative specimen at SIB [074033], which excellently demonstrates the main specific diagnostic traits and agrees with Schur’s description, we designate here as a lectotype of *C.subcorymbosum*, considering the rest (CL [91643], SIB [Herb. Fuss 25.312; Herb. Ungar 42.521]) as isolectotypes.

The heterotypic synonym Pyrethrumcorymbosumf.macrocephalum was published by Waisbecker (1891) accompanied by a short diagnosis (“kétszerte nagyobb fészkekkel [with capitulae twice as large]”) comparing this form to the type, and the citation of two localities (“a Satzenriegel sziklás kupján Rh. és a Kienh. tetején Bkö [on the rocky hilltop Satzenriegel/Satzenstein near Rohonc/Rechnitz and on the top of Kienhegy/Kienberg near Borostyánkő/Bernstein]”). Our attempt to find any original material of the name of this form was in vain. Interestingly, the available later collections of *Tanacetumcorymbosum* s.l. by Waisbecker after 1891 (including those from the region mentioned in the protologue) at herbaria BP and SAMU do not contain any identifications associated with P.corymbosumf.macrocephalum. It is very likely that Waisbecker (1891) described his P.corymbosumf.macrocephalum using specimens of *Chrysanthemumsubcorymbosum* sensu Schur (i.e., *Tanacetumclusii*), and only after his publication was he informed about the description of Schur (1859). That is probably why he labeled similar plants from 1892 onwards as Chrysanthemumcorymbosumvar.subcorymbosum. One of those specimens (SAMU [Herb. Waisbecker]), which is *T.clusii* and was collected by Waisbecker on Ochsenriegel Mt (Appendix 1) in the nearest vicinity of one of the localities cited in the protologue (Kienberg Mt), we designate here as a neotype of P.corymbosumf.macrocephalum.

## ﻿Comments on the distribution

*Tanacetumclusii* is commonly regarded to be distributed in the Eastern Alps and the Carpathians, as well as in the Dinarides (Hegi 1929, as Chrysanthemumcorymbosumvar.subcorymbosum; Piękoś 1971, as Tanacetumcorymbosumsubsp.clusii; Zelený 2004, as *Pyrethrumclusii*). Its occurrence in the Western Alps is doubtful (Meusel and Jäger 1992). The diploid *T.clusii* is found in unglaciated areas and its populations are often disjunct, relict and sometimes prealpine. This is in contrast to the more widespread tetraploid *T.corymbosum*, which is believed to have evolved from the diploid *T.clusii* (Ehrendorfer 1970; Niklfeld 1972; Zimmermann 1972; Nève and Verlaque 2010, as T.corymbosumsubsp.clusii). *Tanacetumclusii* occurs in the montane and subalpine zone at an altitude of 500–2000 m. It grows in open forests, forest edges, megaphorbs and herb-rich meadows on nutrient-rich or sometimes podzolic substrates (Hegi 1929, as C.corymbosumvar.subcorymbosum; Zimmermann 1972; Fischer et al. 2008; as T.corymbosumsubsp.subcorymbosum; Dubyna et al. 2019, as *P.clusii*; Pulišová et al. 2021). It is considered to be a diagnostic species for the *Carpino-Fagetea sylvaticae* and *Mulgedio-Aconitetea* phytosociological classes (Electronic Appendix S6 in Mucina et al. 2016, as T.corymbosumsubsp.subcorymbosum).

The species is currently recorded for Italy (regions: Lombardy, Trentino-Alto Adige, Friuli-Venezia Giulia; appendix S2 in Bartolucci et al. 2018; appendix S1 in Bartolucci et al. 2024; both as Tanacetumcorymbosumsubsp.subcorymbosum), Austria (federal states: Salzburg, Carinthia, Styria, Lower Austria, Burgenland; Jávorka 1924–1925, as *Chrysanthemumclusii*; Fischer et al. 2008; Gilli et al. 2020; as T.corymbosumsubsp.subcorymbosum), Slovenia (Martinčič et al. 2007, as T.corymbosumsubsp.clusii), Croatia (counties: Primorje-Gorski Kotar, Krapina-Zagorje; Nikolić 2020, 2024, as T.corymbosumsubsp.clusii), Poland (voivodeships: Lesser Poland, Subcarpathian; Zając and Zając 2001, as T.corymbosumsubsp.clusii), Slovakia (regions: Žilina, Banská Bystrica, Prešov, Košice; Pulišová et al. 2021; Kochjarová 2023), Ukraine (regions: Lviv, Transcarpathia, Ivano-Frankivsk, Chernivtsi; Dobrochayeva 1962; Chopyk and Fedoronchuk 2015; as *Pyrethrumclusii*), Romania (counties: Maramureș, Bistriţa-Năsăud, Suceava, Cluj, Mureș, Harghita, Covasna, Brașov, Sibiu, Hunedoara, Caraș-Severin, Vâlcea, Olt, Argeș, Prahova, Buzău; Nyárády 1964, as Chrysanthemumcorymbosumvar.clusii; Oprea 2005, as T.corymbosumsubsp.subcorymbosum).

In the botanical literature of the 20^th^ century, there are several mentions of *Tanacetumclusii* for the flora of Switzerland (Canton of Ticino – Chenevard 1910; Schinz and Keller 1914; Hegi 1929; as Chrysanthemumcorymbosumvar.subcorymbosum; Heß et al. 1972, as *C.subcorymbosum*; Val Poschiavo, Canton of the Grisons – Meusel and Jäger 1992), Hungary (Bükk Mts, Northern Hungary; Soó 1970, as C.corymbosumsubsp.clusii), as well as of Bosnia and Herzegovina (entities: the Federation of Bosnia and Herzegovina, Republika Srpska; Maly 1904; Prodán 1910; as C.corymbosumvar.subcorymbosum; Hayek 1928–1931, as T.corymbosumsubvar.subcorymbosum). New sources, however, do not distinguish this species in the above countries (Switzerland – Aeschimann et al. 2004; Juillerat et al. 2017; Eggenberg et al. 2022; Lauber et al. 2024; Hungary – Király 2009; Bartha et al. 2015; Bosnia and Herzegovina – Beck-Mannagetta et al. 1983; Stupar et al. 2021).

At the same time, the distribution of *Tanacetumclusii* in Hungary and in Switzerland is confirmed with the herbarium specimens examined (Appendix 1). At the herbaria BP and DE, we revealed the specimens corresponding to the Bükk mountain range mentioned in the above published source (Soó 1970). Additionally, at the herbaria BP and W, we found specimens collected in the Mátra Mts, which, along with the nearby Bükk Mts, are the sections of the Inner Western Carpathians in Hungary. Moreover, at the herbarium SAMU, there are several specimens collected in the Kőszeg Mts representing the easternmost part of the Eastern Alps. Given that the published available data for the flora of Hungary (Soó 1970), only mentioned the occurrence of *T.clusii* in the Bükk Mts, the above records from Mátra and Kőszeg ranges are new findings for Hungary and confirm a broader distribution of the species in this country.

At the herbarium ZT we revealed specimens of *Tanacetumclusii* collected in Puschlav/Val Poschiavo, Canton of the Grisons in Switzerland (Appendix 1), confirming the statement in Meusel and Jäger (1992). The single record for the Canton of Ticino is based on a herbarium voucher collected in Morbio super[iore] (Chenevard 1910; as Chrysanthemumcorymbosumvar.subcorymbosum). Chenevard’s statement about the size of capitula being the same as in C.corymbosumvar.corymbosum (“mais à capitules de même grosseur que ceux du type”) is already a hint for a misidentification. Moreover, the respective specimen cited by Chenevard (1910): SWITZERLAND. Ticino: Près à Morbio super (Tessin), 18 Jun 1905, *Giulietti s.n.* (G [G00382124!, Herb. Chenevard]), cannot be assigned to *T.clusii*. This and some other collections from the Southern and Western Alps are rather approaching T.corymbosumsubsp.achilleae (L.) Greuter (Iamonico 2018) and require a detailed revision (see also Tison and de Foucault 2014, pg. 394). We therefore regard the Canton of the Grisons, Switzerland and the province of Brescia in the Lombardy region, Italy (Martini et al. 2012) as the westernmost occurrences of *T.clusii*.

Regarding Bosnia and Hercegovina, we recognize the occurrences of *Tanacetumclusii* provided in the older botanical literature and highlight the necessity of confirming these data with new field research.

*Tanacetumclusii* is also reported as introduced (POWO 2024, as T.corymbosumsubsp.subcorymbosum) or doubtfully native (Euro+Med 2024, as T.corymbosumsubsp.subcorymbosum) in France, where it is recorded from Var department in the Provence-Alpes-Côte d’Azur region (Charpin and Salanon 1988, as T.corymbosumsubsp.clusii; Iamonico 2018, as T.corymbosumsubsp.subcorymbosum; Tela Botanica 2024, as T.corymbosumvar.subcorymbosum). We consider this information erroneous, given that it is based on the taxonomic misinterpretation provided in Charpin and Salanon (1988). With T.corymbosumsubsp.clusii (i.e., *T.clusii*), Charpin and Salanon (1988) synonymized the name T.corymbosumvar.macroglossum Briq. & Cavill. applied for a taxon first described from the Tanneron Massif in the Var department (Briquet and Cavillier 1916). At the same time, in the protologue of T.corymbosumvar.macroglossum (Briquet and Cavillier 1916, pg. 124), this variety was clearly differentiated from T.corymbosumvar.subcorymbosum (Schur) Simonk. (i.e., *T.clusii*) not present in the studied territory. Notably, the involucral bracts in T.corymbosumvar.macroglossum were reported as edged pale brown, being brownish at the apex. The type material of this variety couldn’t be found in G and is probably lost, corresponding to its status “à rechercher [to be searched]” provided by Briquet and Cavillier (1916). Current sources consider T.corymbosumvar.macroglossum a taxonomic synonym of *T.corymbosum* s.str., and not of *T.clusii* (Hassler 2024; POWO 2024; both as a synonym of T.corymbosumsubsp.corymbosum). This is also a probable reason why *T.clusii* is not mentioned at all in the most authoritative current works on the flora of France (Tison and de Foucault 2014; Tison et al. 2014).

Some authors (Verlaque and Contandriopoulos 1990; Nève and Verlaque 2010; referring to it as Tanacetumcorymbosumsubsp.clusii) stated that the distribution of *T.clusii* extends to the Rhodopes, a mountain range in Bulgaria and Greece. However, this is probably referring to T.corymbosumsubsp.cinereum (Iamonico 2018; Euro+Med 2024). Such reports of *T.clusii* appear to be erroneous, as they are not supported by any historical and contemporary floristic data for Bulgaria (e.g., Velenovský 1891; Hayek 1928–1931; Assyov et al. 2012; Kuzmanov 2012; Stoyanov et al. 2021) and Greece (e.g., Halácsy 1902; Dimopoulos et al. 2013, 2016; K. Tan, personal communication). Similarly, the Euro+Med PlantBase (Euro+Med 2024, as T.corymbosumsubsp.subcorymbosum) considers this species native to Turkey, citing Güner et al. (2012) as a source of this statement. Given the absence of any mention of *T.clusii*, including under any of its synonymic names in Güner et al. (2012), it seems there is a technical error in Euro+Med (2024). Moreover, the species is not cited in any primary sources concerning the flora of Turkey (e.g., Grierson 1975; Bizim Bitkiler 2024). Another technical error seems to have occurred in Meusel and Jäger (1992), where this species was reported in Montenegro. This is supported by the fact that these data were not included on the respective map in this work. Additionally, there are no records of *T.clusii* in the fundamental works on the flora of Montenegro (e.g., Rohlena 1942; Pulević 2005). Moreover, this species was previously recorded from the Czech Republic (Dostál 1989, as *Pyrethrumclusii*), which was later considered to be erroneous (Zelený 2004; Euro+Med 2024).

In summary, the native range of *Tanacetumclusii* encompasses Switzerland, Italy, Austria, Slovenia, Croatia, Bosnia and Herzegovina, Hungary, Slovakia, Poland, Ukraine, and Romania. At the same time, the reported presence of *T.clusii* in Bulgaria, the Czech Republic, France, Greece, Montenegro and Turkey has not been confirmed.

## Supplementary Material

XML Treatment for Tanacetum clusii

## ﻿Additional information

### Author ORCIDs

Viktor O. Nachychko https://orcid.org/0000-0001-6756-2823

Clemens Pachschwöll https://orcid.org/0000-0002-8281-7545

Mihai Puşcaş https://orcid.org/0000-0002-2632-640X

Gergely Király https://orcid.org/0000-0002-8439-2616

### Data availability

All of the data that support the findings of this study are available in the main text.

## ﻿Appendix 1. The studied specimens of *Tanacetumclusii*

**Authentic and type material**

Austria. **Lower Austria**: Furth bei Weissenbach a. d. Triesting, Population in Wiese, 2 Jun 1972, *B. Drescher s.n.* (WU [WU0153863!, image available at https://wu.jacq.org/WU0153863; WU0153864!, image available at https://wu.jacq.org/WU0153864; WU0153865!, image available at https://wu.jacq.org/WU0153865; WU0153866!, image available at https://wu.jacq.org/WU0153866; WU0153867!, image available at https://wu.jacq.org/WU0153867; WU0153868!, image available at https://wu.jacq.org/WU0153868; WU0153869!, image available at https://wu.jacq.org/WU0153869; WU0153870!, image available at https://wu.jacq.org/WU0153870]); **Burgenland**: Az Ökörgerincz hegyen Vörösvágás mellett [on the Ochsenriegel Mt close to Redlschlag], 20 Jul 1892, *A. Waisbecker AW 755* (SAMU [Herb. Waisbecker!]). ROMANIA. **Brașov**: In monte calcareo Koenigstein [Piatra Craiului Mts] prope Coronam [Brașov], Aug 1853, *F. Schur s.n.* (LW [LW00208453!]); In fruticetis montanum calcareanum prope Coronam [Brașov], Jul 1854, *F. Schur s.n.* (LW [LW00208447!]); **Brașov/Dâmbovița/Prahova**: In monte calcareo Butsets [Bucegi Mts], 7000’, Aug 1854, *F. Schur s.n.* (LW [LW00208452!]); **Harghita**: Öcsém [Piatra Ascuțită/Ecem], 29 Jul [1853], *F. Schur s.n.* (SIB [Herb. Ungar 42.521!]); Oecsém [Piatra Ascuțită/Ecem], [1853], *F. Schur s.n.* (SIB [Herb. Fuss 25.312!]); In monte calcareo Ecsém Tetei [Piatra Ascuțită/Ecem], 1853, *F. Schur s.n.* (SIB [074033!]); In monte calcareo Ecsém Tetei [Piatra Ascuțită/Ecem], 29 Jul 1853, *F. Schur s.n.* (CL [91643!]).

**Specimens confirming the species distribution in some countries**

Switzerland. **Grisons**: Puschlav, Valle Cologna, 1400 m, 20 Jun 1904, *H. Brockmann-Jerosch s.n.* (ZT [ZT-00283034!, image available at https://www.digitalis.uzh.ch/media/specimen/283/ZT-00283034]); Puschlav, Buschweide ob Prada, im Gebüsch, S-W, 1120 m, s. dat., *H. Brockmann-Jerosch A247* (ZT [ZT-00283036!, image available at https://www.digitalis.uzh.ch/media/specimen/283/ZT-00283036]); Im Lindenbuschwald am Puschlavsee [Lago di Poschiavo] am SW-Hang bei Meschino [Mota dal Meschin], 7 Aug 1944, *W. Trepp-Fredenhagen s.n.* (ZT [two sheets: ZT-00283030!, image available at https://www.digitalis.uzh.ch/media/specimen/283/ZT-00283030; ZT-00283031!, image available at https://www.digitalis.uzh.ch/media/specimen/283/ZT-00283031]). HUNGARY. **Western Transdanubia, Vas**: A felső erdőben Kőszegen, 24 Jun 1895, *A. Waisbecker s.n.* (SAMU [Herb. Waisbecker!]); In silvis caeduis ad Velem, Langer Graben [Hosszú-völgy], 500 m, solo phyllitico, 25 Jul 1899, *V. Piers s.n.* (SAMU [Herb. Piers!]); Langer Graben [Hosszú-völgy], 25 Jul 1899, *V. Piers s.n.* (SAMU [Herb. Piers!]); **Northern Hungary, Borsod-Abaúj-Zemplén**: Mt. Bükk, in decliviibus montis “Odorvár” supra pag. Cserépfalu, 17 May 1983, *D. Kováts s.n.* (BP [017116!]); **Heves**: Bükk hegység, Szalajka-völgy, Erdei vágásban, 10 Jul 1954, *Z. Siroki s.n.* (DE!); Bükk hegység, Gerennavár, tölgyerdőben, 22 Jun 1960, *Z. Siroki s.n.* (DE!); In rupibus andesiticis umbrosis montis Saskő pr. Parádfürdő, 8[00]–900 m, 5 Aug 1928, *Á. Boros s.n.* (BP [463441!]); Matragebirge, Weg vom Sas-kő zum Disznó-kő, ca. 800–900 m; Buchenwald, Andesit, 10 Jul 1973, *E. Krendl & F. Krendl s.n.* (W [W0215240!, image available at https://w.jacq.org/W0215240]).
